# High predictive values of RBC membrane-based diagnostics by biophotonics in an integrated approach for Autism Spectrum Disorders

**DOI:** 10.1038/s41598-017-10361-7

**Published:** 2017-08-29

**Authors:** Giorgia Giacometti, Carla Ferreri, Anna Sansone, Chryssostomos Chatgilialoglu, Carla Marzetti, Ellas Spyratou, Alexandros G. Georgakilas, Marina Marini, Provvidenza M. Abruzzo, Alessandra Bolotta, Alessandro Ghezzo, Renato Minguzzi, Annio Posar, Paola Visconti

**Affiliations:** 1 ISOF, Consiglio Nazionale delle Ricerche, Via P. Gobetti 101, 40129 Bologna, Italy; 2 Laboratorio Valsambro Srl, Via Cairoli 2, 40121 Bologna, Italy; 3 Physics Department, School of Applied Mathematical and Physical Sciences, National Technical University of Athens, Zografou Campus, 15780 Athens, Greece; 4 Department of Experimental, Diagnostic and Specialty Medicine, School of Medicine, University of Bologna, Via Belmeloro 8, 40126 Bologna, Italy; 5 Don Carlo Gnocchi Foundation ONLUS, IRCCS “S. Maria Nascente”, Via Alfonso Capecelatro 66, 20148 Milan, Italy; 6 Comune di Bologna, Piazza Liber Paradisus 10, 40129 Bologna, Italy; 7 Department of Biomedical and Neuromotor Sciences, University of Bologna, Via Altura 3, 40139 Bologna, Italy; 8 IRCSS Institute of Neurological Sciences of Bologna, Child Neurology Unit, Via Altura 3, 40139 Bologna, Italy

## Abstract

Membranes attract attention in medicine, concerning lipidome composition and fatty acid correlation with neurological diseases. Hyperspectral dark field microscopy (HDFM), a biophotonic imaging using reflectance spectra, provides accurate characterization of healthy adult RBC identifying a library of 8 spectral end-members. Here we report hyperspectral RBC imaging in children affected by Autism Spectrum Disorder (ASD) (n = 21) compared to healthy age-matched subjects (n = 20), investigating if statistically significant differences in their HDFM spectra exist, that can comprehensively map a membrane impairment involved in disease. A significant difference concerning one end-member (spectrum 4) was found (*P* value = 0.0021). A thorough statistical treatment evidenced: i) diagnostic performance by the receiving operators curve (ROC) analysis, with cut-offs and very high predictive values (*P* value = 0.0008) of spectrum 4 for identifying disease; ii) significant correlations of spectrum 4 with clinical parameters and with the RBC membrane deficit of the omega-3 docosahexaenoic acid (DHA) in ASD patients; iii) by principal component analysis, very high affinity values of spectrum 4 to the factor that combines behavioural parameters and the variable “cc” discriminating cases and controls. These results foresee the use of biophotonic methodologies in ASD diagnostic panels combining with molecular elements for a correct neuronal growth.

## Introduction

Membranes constitute an interesting observational site to get molecular information in various health conditions. Not only the structural disposition of protein channels and lipid components influencing fluidity and permeability, but also the presence of specific fatty acid components in membrane phospholipids, involving receptor functioning and intra/inter cellular signaling, provide proper conditions for physiological operations^1–6^. Considering that blood transports essential nutrients to the brain, blood-based biomarkers, reporting the molecular status obtained from stabilized dietary conditions, are used to assess risk factors in several neurological and cognitive diseases^1, 5^. Data from our and others’ laboratories pointed attention to fatty acid unbalances of red blood cell (RBC) membrane lipidome, as well as to morphological defects in autism spectrum disorders (ASD), influencing cell growth and functions^7–9^. Interestingly, in autistic patients an unbalanced fatty acid content and poor functionality of Na/K pump in RBC membranes were found, being several fatty acids also correlated to the severity of behavioural and cognitive scores^7^. Regarding these types of deficit in patients and the use of omega-3 supplements in autism, there is an active debate on positive results obtained in some studies, but more insights are needed^10–12^. It is remarkable that so far ASD subjects have never been controlled by RBC membrane lipidomic analysis before and after supplementation. Another interesting result of investigations is that high-risk infants, that later are diagnosed with ASD, show evident atypical brain development and function within the first 12 months with neurobiological signs^13^. Being ASD a multi-factorial disorder, with complex interactions between genetic and environmental risk factors^14^, research advances on comprehensive diagnostic tools are needed, in order to develop integrated panels taking also into account essential elements from nutrition, that are necessary for a correct child growth.

Hyperspectral dark field microscopy (HDFM) is an emerging biophotonic imaging methodology using visible light to get a very accurate digital image description, by acquisition of hundreds of pixels, at a pixel size of 25 nm and spectral resolution of 2.0 nm, of the diffusely- scattered light, *i*.*e*., reflectance, in the 400–1000 nm range. Spectral reflectance is gathered for each pixel and leads to the individuation of very detailed and informative scattering spectra referred to the sample characteristics. Assigning a color code to the spectral end-members identified in the sample, the optical image is converted to a false-colored spectral imaging, utilizing many color channels, and the satisfactory match between optical and spectral data can be visualized. Spectral bands are collected in spectra of different intensities that are characteristic of each observed sample or object. According to the sample type, standard reference spectral libraries can be built-up, useful to examine the component fingerprints in samples under different conditions. As matter of facts, HDFM spectral analysis can compare pixel spectra with a reference spectrum, and the spectrum of a single component can be used in order to understand the relevance of this component in a complex sample. Using spectral classification map and their percentage distribution, created by the spectral mapping software, the HDFM spectral features are precisely described, therefore changes occurring in the samples are reflected in changes of HDFM spectral distribution^15, 16^. We focused on HDFM for the comprehensive and advanced imaging of complex biological scenarios such as human red blood cell (RBC) membrane^17^, and first published the healthy adult RBC imaging by a library of 8 spectral end-members with their typical distribution map^18^. In the RBC membrane and sub-membrane regions, cytoskeleton proteins, phospholipids and membrane-bound hemoglobin are the main constituents. The HDFM spectra are comprehensive descriptors of thickness, shape, refractive index, anisotropy and geometry of the membrane compartment and, at molecular level, describe the structures and properties of the light-intercepting components. On these premises, we were interested in the HDFM potentiality to image different RBC membrane molecular status that could be correlated to the health condition. We thought to examine RBC in ASD, a disease previously studied by us for membrane fatty acid content^7^, with the aim at investigating if statistically significant differences in HDFM spectra exist comparing ASD and healthy children.

Here we report the results of hyperspectral RBC imaging obtained from a cohort of ASD children compared to healthy age-matched subjects. In our membrane-based diagnostic approach, a parallel fatty acid analysis for all subjects was considered, in order to correlate data of these RBC membrane molecular components with the HDFM analyses. A thorough statistical treatment was carried out, in order to individuate significant differences between the two sets of data, and to evaluate how the combination of spectral and molecular information can provide cut-offs and high predictive values for the individuation of ASD patients within the children cohorts. The general aim of this work is to contribute to the investigation of multifactorial health conditions by membrane-based diagnostic approach, which can be complementary to genetic and epigenetic research in ASD.

## Results

### HDFM spectra from healthy and ASD children are significantly different and with high predictive value for the disease

**Figure 1:**
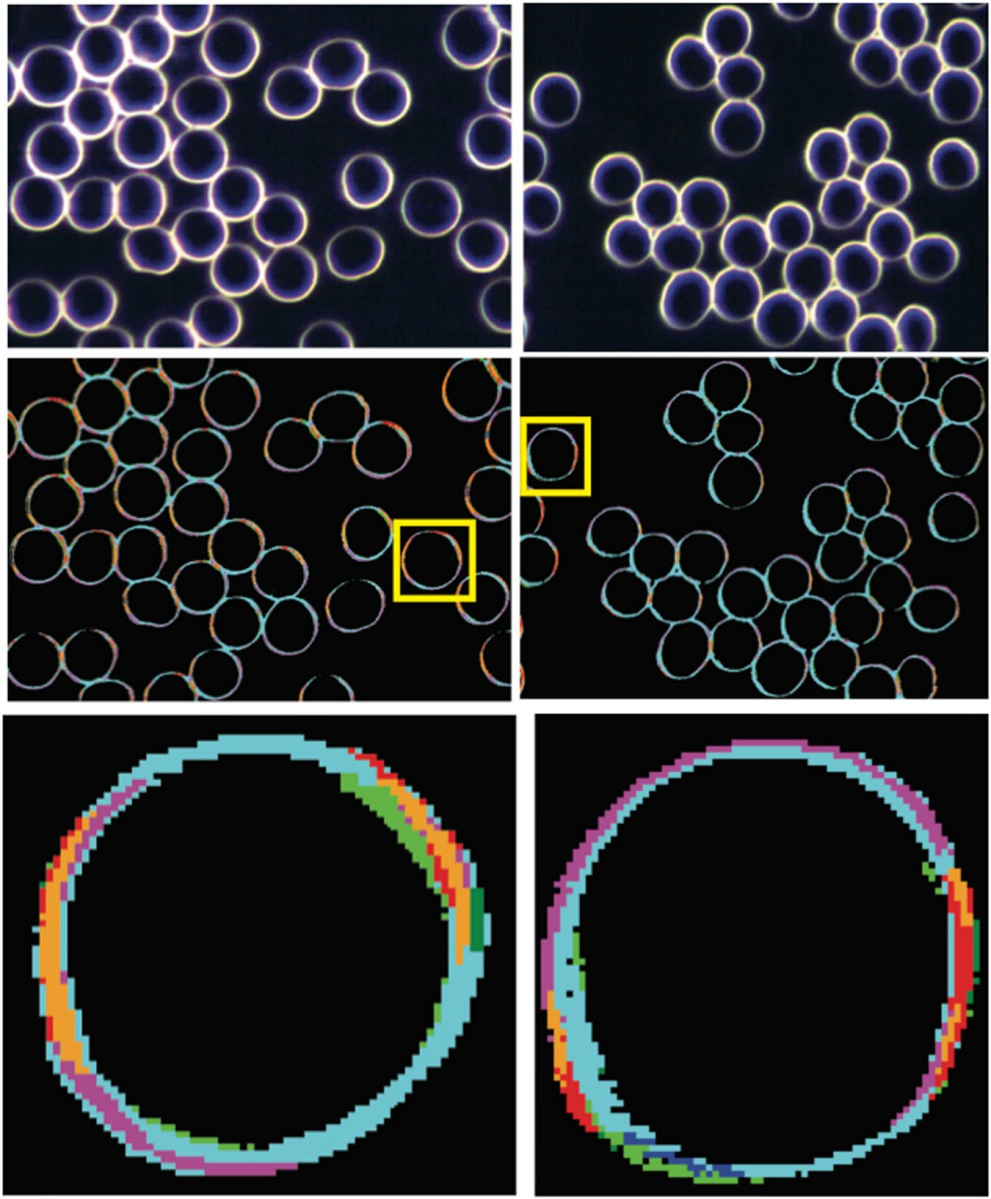
RBCs optical images and the corresponding hyperspectral image for healthy (left panels) and ASD (right panels) children. In yellow box a selected RBC for which an enlarged view is given below, where it is clearly seen the satisfactory matching of the optical and hyperspectral images. Coloured areas indicate regions whose reflectance spectra match with the spectral end-members of the library, as shown in Supplementary Fig. 1.

A total of 41 children were recruited, 21 with ASD (15 Males and 6 Females, mean age 7.2 ± 0.8 yrs) and 20 with a typical development (13 Males and 7 Females, mean age 9.0 ± 0.9 yrs). Patients were admitted to the Child Neuropsychiatry Unit of the Bellaria Hospital (IRCCS, Bologna) for a clinical diagnostic assessment and a comprehensive neurological work up. Details of recruitment and diagnostic panel are described in the Methods. In all 21 patients medical, genetic and neurological comorbidity was excluded. Childhood Autism Rating Scale (CARS) total scores ranged from mild to severe autistic features, while developmental assessments showed variability from normal IQ to severe cognitive impairment (Supplementary Table 1). A small sample of fresh whole blood (500 μL), treated with ethylenediaminetetraacetic acid (EDTA) as the anticoagulant, was collected from all subjects and used for HDFM measurements and membrane lipidome analyses. Equipment settings and handling protocol for hyperspectral imaging are reported in Methods. Optical images of RBC were acquired for all samples, and for uniformity of the sampling procedure, we choose to have the prevalent RBC round shape in the optical field, excluding echinocyte and stomatocyte forms, which are known to be increased in autism^7, 8^, but can also depend from pH and other blood parameters, or even be artefacts during blood storage or oxidative transformations. Accurate collection of the spectral reflectance from RBCs gave rise to 8 spectra in the 430–800 nm wavelength region (Supplementary Fig. 1). It is worth noting that, using a small spectral angle (0.1 in radians), the 8 spectra satisfactorily gave a fingerprint of the Region of Interest (ROI), with optimal coverage of the optical image (>98%) as shown in Fig. 1 (see Supplementary Figure 1 – panel A – for the original HDFM spectra before the smoothing procedure). We were also aware that the cell membrane curvature, determined by the orientation of the blood cells relative to the propagation vector of the illuminating electro-magnetic field, could have a significant influence. When a biconcave shaped RBC interacts with a linearly polarised light beam tends to orient its long axis in the direction of the electric field of the incident beam, due to RBC’s intrinsic birefringence. In our case, the light source was an unpolarized halogen light source, so no “forced” orientation of the cells was observed. The lamp normalization routine was used in order to eliminate the background light, ameliorating the results obtained by the method previously reported by us^17^. Under these careful set-up conditions, we constantly obtained a good match by mapping of the 8 end-members spectral library with the small spectral angle onto images taken from different blood samples. Therefore, we could conclude that the identified spectral signatures are not sensitively affected by the cell orientation, but probably result from a particular curvature of the membrane surface connected to the morphological status. Indeed, the cell orientation dependency could be observed when differently shaped RBCs are examined, such as stacked RBCs forming roleaux and echinocytes, giving rise to different spectral end-members^17^, that are currently under investigation for the biological importance of these changes.

**Figure 2:**
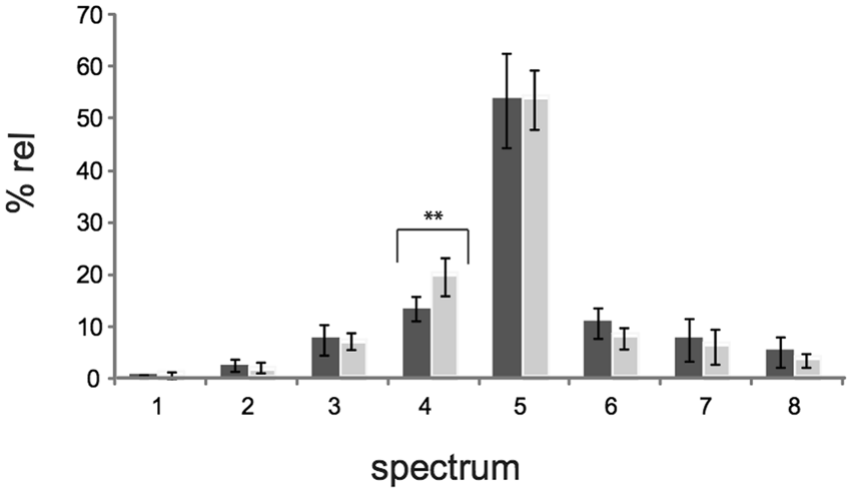
Histograms of distribution (%rel ± SEM) of the 8 spectral end-members of HDFM library for RBC imaging as obtained by the Spectral Angle Mapper (SAM) software of the hyperspectral microscope. Spectral distribution is obtained by the SAM function in RBCs of healthy (dark grey, n = 20) and ASD (light grey, n = 21) children and data are reported in Supplementary Table 2.

By using the Spectral Angle Mapper (SAM) software the relative spectral distribution in hyperspectral images of all samples could be obtained. Mean distribution values with standard errors for the two children cohorts are reported as histograms in Fig. 2 and data are shown in Supplementary Table 2. Actually, the distribution data of the ASD children cohort showed high variability for four out of the eight spectra, therefore in Supplementary Table 2 only four spectra are shown which did not have large errors in their distribution percentages. By analysis of variance (ANOVA, see details in Methods) the spectrum 4 distribution resulted significantly different between healthy and ASD children (*P* value = 0.0021). Statistical analysis for the diagnostic performance of spectrum 4 distribution values was carried out using the receiver operating characteristic (ROC) curve analysis^19^. Very high statistical significance (*P* value = 0.0008) and a cut-off value at 16.225 were determined. Together with the odds ratio, found to be significant and corresponding to 24 (*P* value < 0.0001; IC 95%: [4.6488–123.9035]), statistics evidenced that individuals with distribution values of spectrum 4 higher than 16.225 (cut-off value) have a probability of being autistic 24 times higher than those having values of spectrum 4 lower than 16.225 (see Methods and Supplementary Information for more details on statistics).

**Figure 3:**
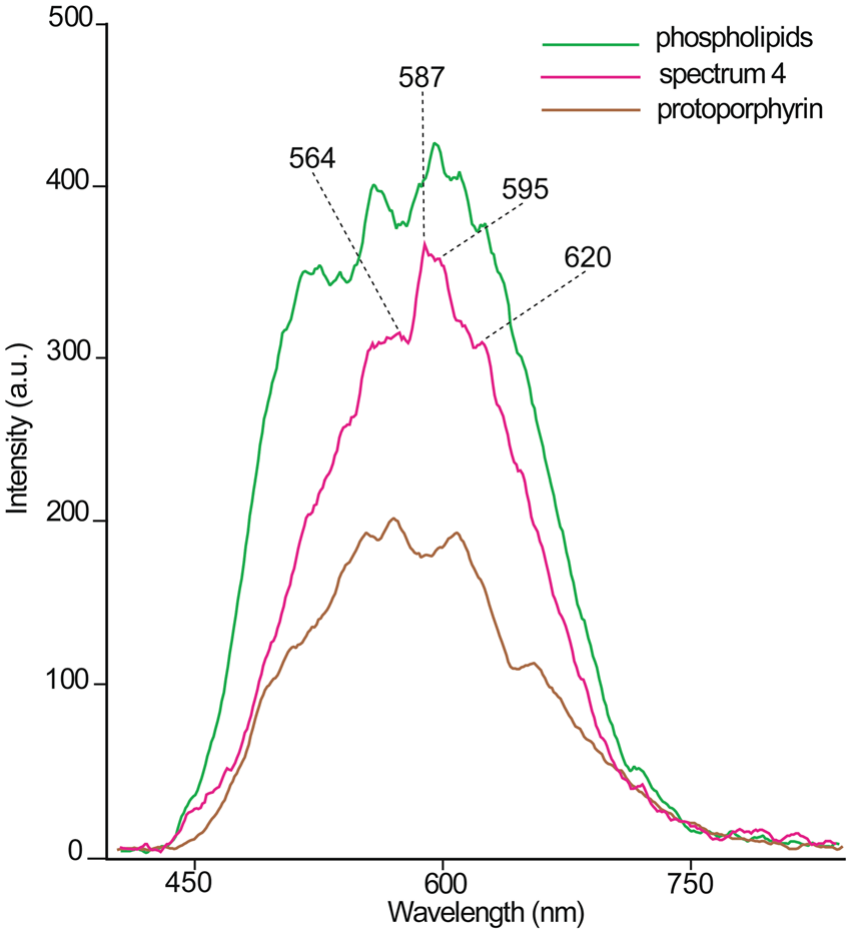
HDFM spectrum 4 in the 400–800 nm wavelength range and superimposition with the HDFM spectra of two reference compounds, phospholipids as liposome suspension (green) and protoporphyrin IX (brown), separately acquired as previously described^18^ and reported in Supplementary Information. Principal bands of the spectrum 4 are indicated.

Figure 3 shows the end-member 4 and its spectral features with bands at 564, 587, 595, 620 nm. In the same Fig. 3 the HDFM spectra registered for phospholipids and protoporphyrin IX, which are important components of the RBC membrane and sub-membrane regions, are shown. The acquisition of these spectra was carried out as previously reported^18^ (see also details in the Supplementary Information). In Supplementary Fig. 1 (panel B) their original HDFM spectra before the smoothing procedure are shown. It is worth underlining that the phospholipid sample was prepared as aqueous liposome suspension^18^, an aggregation that mimics the membrane lipid organization. Some of the spectral bands in common between spectrum 4 and the two membrane components can be seen. Further work is needed to make an unequivocal assignment of the spectral bands in RBCs that is not the scope of this report.

Examining the HDFM spectral end-member distribution in healthy children and in particular the spectrum 4 in comparison with the previously collected data on healthy adults^18^ (cfr., Supplementary Tables 2 and 3), it was gratifying to see that no consistent difference occurs. Indeed, statistical significance and cut-off value for individuating ASD children, as above indicated, were confirmed over the total group of adult and young subjects (data not shown). Finally, Spearman’s non-parametric correlations were calculated for HDFM data and clinical features of ASD children. Again, the spectrum 4 was found to be positively and significantly correlated with CARS total scores (*P* = 0.0248), hyperactivity (*P* = 0.0279), stereotypies (*P* = 0.0225) (Supplementary Figs 3–5).

### DHA decrease in RBC membrane phospholipids increases the predictive value for ASD when associates with HDFM data

An aliquot of the same blood sample (250 μL) of healthy and ASD children was processed for fatty acid analysis of membrane phospholipids, in order to evaluate statistically these results for the first time in combination with the HDFM data. The RBC membrane fatty acids of healthy and ASD subjects are reported in Supplementary Table 4, evidencing a significant DHA decrease in disease (*P* value = 0.0424), as already reported for previously studied cohorts^7, 8^. Using ANOVA test (see **Methods** and Supplementary Table 5S) to compare the two groups, the DHA decrease in ASD was found to be significant (*P* value = 0.0344). This decrease was not attributable to dietary differences between the two groups, as evaluated by food questionnaire indicating, for example, fish consumption (data not shown). Statistical significance test of ROC curve for DHA (*P* value = 0.0424) with a cut-off value at 4.08% gave a significant odds ratio corresponding to 6.23 (*P* value = 0.017; IC 95%: [1.3956–27.8412]), which means that individuals with values of DHA < 4.08% (cut-off) have a probability of being autistic 6.23 times higher than those with DHA > 4.08% (Supplementary Fig. 6). More interestingly, the combination of ROC curves of HDFM spectrum 4 and DHA (Supplementary Fig. 7) gave a higher significance, also greater than DHA alone (*P* value = 0.0004 *vs*. *P* value = 0.0424), with a still strongly significant odds ratio (*P* value = 0.002; IC 95%: [2.65624–80.52379]). The value of the combined odds ratio (14.625) is not considered, remaining the odds ratio of 24 and 6.23 to express the diagnostic performance of spectrum 4 and DHA, respectively. By statistical treatment of all data using the principal component analysis (PCA), four main factors were individuated. Interestingly, the parameters of DHA, behavior (CARS), spectrum 4 and the variable that discriminates cases from controls (“cc”) are in the same Factor (Factor 2) with good factorial weights, that express the affinity of these variables (see Supplementary Information Fig. 8 and related tabulation of the factorial weights, grey color). The negative DHA factorial weight means that this parameter is higher when the values of the other variables, belonging to the same factor, are lower. Some other results emerged, such as the very high affinity values of Spectra 1 and 2 present in Factor 4, that is represented almost entirely by these two parameters, thus suggesting the PCA treatment as an efficient tool for examining population data.

## Discussion

Biophotonic imaging by HDFM was already known for its ability to distinguish between healthy and unhealthy conditions in tissues^20–22^. An exhaustive description of the hyperspectral imaging (HSI) system with the literature on medical applications can be found^22^. Having reported the HDFM spectral library of healthy adult RBCs^18^, it was interesting to examine if changes occur in different age and disease conditions. The healthy children group (n = 20) was useful for a first comparison with healthy adults (cfr., Supplementary Tables 2 and 3), verifying that HDFM data do not greatly suffer from subject’s age. On the other hand, as shown in Fig. 2 and Supplementary Tables 2 and 3, the distribution percentages of the HDFM spectra are quite different in the healthy and unhealthy children cohorts. Spectra also display low or high intensities, which are known to depend from several factors, such as for example the pigment packaging^23^. It should be underlined that each HDFM end-members has its spectral distribution value (±errors), expressed as percentage of the total scattered light. In the ASD cohort the relative percentage of four spectra, namely spectra 1, 2, 7, 8 (see Fig. 2), had a large variability, sometimes reaching a SEM value equal to 100% of the mean value, and accounted for about a 12–15% of total scattered light (see Supplementary Table 2). On the other hand, the other four HDFM spectra, namely spectra 3, 4, 5, 6, had small errors and among them the spectrum 4 was the one considered for its diagnostic value in this study. It is worth underlining that the ASD subjects in this study had a large clinical heterogeneity (see Supplementary Table 1). We believe that enlarging the study to a large population in ASD will give support and detail better the importance of the eight HDFM end-members individuated for healthy subjects.

Looking closely to the significant data in the ASD and healthy groups, it was gratifying to see that the spectrum 4 in ASD subjects has a distribution score of 20 ± 3, that does not overlap neither with healthy children (13 ± 2) nor with healthy adult groups (12 ± 2). This renders the spectrum 4 a strong parameter to be evaluated in a blind procedure for its discriminating power to distinguish healthy and unhealthy subjects. ANOVA test for spectrum 4 gave a *P* value of 0.0021 (see **Methods** and Supplementary Table 5) and the ROC curve gave a clear-cut indication that this parameter is able to predict a specific subject as healthy (negative group) or affected by ASD (positive group), using the cut-off value of 16.225 for its spectral distribution in the RBC HDFM imaging. Moreover, a series of clinical features were evaluated by non-parametric correlation method (see **Methods**), and the HDFM spectrum 4 correlated positively with increased impairments of behavior and cognition scores (see Supplementary Figs 3–5). In Fig. 3 the spectrum 4 is reported together with the HDFM spectra of two relevant components of the RBC membrane and sub-membrane regions, phospholipids and protoporphyrin IX. It is worth noting that the phospholipid sample was made of egg lecithin prepared as an aqueous liposome suspension^18^, therefore its scattering properties refers to the phospholipid double layer that is the best mimics of biological membrane organization. Although the spectra are broad, similarities between these spectra and spectrum 4 in Fig. 3 can be appreciated. It is also remarkable that the scattering at 620 nm is already used to identify protein-membrane lipid assembly, such as lipid rafts in RBC after density gradient centrifugation^24^. On the other hand, we cannot exclude that spectral bands from RBC molecular components not considered so far are superimposed with those here described.

The spectral and spatial information obtained by the SAM software at 2 nm resolution and with the maximum angle (radians) at 0.1, comprehensively describe the arrangement and chemical/molecular interactions in the sample, and the system is able to detect small changes at nanoscale level with high sensitivity which would be difficult to examine at once by other means. Indeed, the identification of the spectrum 4 as significant variation in RBC samples of healthy and ASD children is an important discovery that opens new perspectives for the RBC membrane-based diagnostics in this disease. Moreover, hyperspectral microscopic blood imaging is more and more emerging since it can improve the traditional methods of light microscopy observation which are used in hematology but require skilled personnel, such as recently described in acute lymphoblastic leukemia^25^. Since a very reduced manipulation of the whole blood drop is needed, this automated methodology is attractive for large cohort studies. We are aware that more work is needed for determining the potential of HDFM to intercept molecular changes due to diseases and unveil differences among different types of diseases. We foresee that the use of HDFM in population studies will further assess and expand its applicability in ASD diagnostics, offering the possibility to combine this information with other molecular data obtained by –omic approaches.

It is also attractive the fact that membranes are made of fatty acids, which are in their turn components of the nutrition, with the essential omega-6 and omega-3 polyunsaturated fatty acids (PUFA) especially important for the correct child growth. Moreover, fatty acids in membrane phospholipids are evaluated in many diseases, including neurological ones, finding out that RBC membranes give precious indications on omega-3 and other fatty acids that influence brain structures, functions and behavior^26, 27, 28, 29^. Membrane fatty acid analysis in our ASD cohort evidenced a significantly different DHA level (ANOVA test, *P* value = 0.0344). In other children cohorts, several impairments of fatty acid levels were reported by us and others^7, 9, 14, 19^, also involving monounsaturated and polyunsaturated components. In the children cohorts of the present study only DHA level was found significantly different and examination of the food questionnaire clarified that was independent from the food intakes. DHA is a fundamental fatty acid, more specifically than omega-3 fatty acids, in general for neuronal health and protection^1, 30^, lacking in the present and in all previously studied ASD cohorts. This highlights its relevance as a consequential factor. Recently, DHA deficit was also found in coeliac patients and has been correlated to deficiency of the brain-derived neurotrophic factor (BDNF)^31^, a well-known biomarker in mood disorders and autism^32, 33^. It must also be taken into account that a high saturated fat diet reduces both DHA and BDNF levels, as reported in animal models^34^, and that the DHA decrease in RBC membrane of ASD patients could also recall the influence of maternal dietary fats on autism onset^35^. The molecular unbalances detected in RBC membranes can be easily translated into practical guidelines to address the found fatty acid deficit such as DHA with a tailored supplementation, providing specific formulas and not giving generically omega-3 or other unneeded fats. As matter of facts, nutraceutical intervention has been previously reported in autism with omega-3 fatty acids^5, 10–12, 36^ without detailing the complete fatty acid composition of the supplementation. So far the results of nutraceutical intervention are reputed highly controversial^37^, however the lack of personalization, without assessing the individual needs before assigning the supplementation and the bioavailability by incorporation in cell membranes, is a big limiting factor to the success of the nutra-therapy. We are aware of the fact that several nutritional factors have been outlined for their importance in the pathobiology of autism^38^, and the puzzle of ASD influencing factors cannot be limited to lipids, while addressing the deficit of essential components like DHA.

Our results point to a new RBC membrane-based diagnostic approach for the examination of ASD and healthy children by combining biophotonic data with molecular information of fatty acid-based functional lipidomics. In particular: (a) the ROC curve using both the HDFM spectrum 4 and DHA values was built-up and gave very significant results (*P* value = 0.0004, AUC 0.8238, sensitivity 95%, Youden index J = 0.5690, see **Methods** and Supplementary Fig. 7); (b) the cut-off values of spectrum 4 (16.225) and DHA (4.08) were individuated and can provide a good starting point for evaluation of large cohorts; (c) using PCA as statistical methodology, the affinities of multiple parameters was examined and Factor 2 combined good factorial weights of 4 data, namely the HDFM spectrum 4, DHA, CARS clinical score and the parameter “cc”, the latter being the variable that distinguish cases and controls (see Supplementary Fig. 8 and related data tabulation with meaningful values in grey colour). By the PCA statistical method it is interesting that all data obtained from the cohorts are used, and the spectrum 4 was found the only one associated with parameters other than the spectral ones. This statistical treatment gave the best confirmation of the diagnostic value of the spectrum 4 in the RBC HDFM library regarding ASD patients. It is worth mentioning that the PCA method indicates affinities and not correlation between these parameters.

In conclusion, the approach here described suggests biophotonic methodologies as part of a multidisciplinary platform combined with molecular diagnostics, such as lipidomics, to be able to give a more comprehensive description of cell compartments such as RBC membranes. Considering its non-invasiveness together with fastness and reasonable costs, HDFM can be implemented for large cohort studies in ASD, including very young children and siblings^39^, with further possibility to monitor molecular impairments and effects of nutritional strategies for recovering the balance and pursue a correct child growth.

## Methods

### Demographic and clinical features of human subjects

A total of 41 children were recruited, 21 with ASD (15 Males and 6 Females, mean age 7.2 ± 0.8 yrs) and 20 with a typical development (13 Males and 7 Females, mean age 9.0 ± 0.9 yrs). The present study was conducted according to the guidelines laid down in the Declaration of Helsinki and the Ethical Committee of Bologna Health Authority approved all procedures involving human patients (authorization n. 13062). Their parents gave the informed consent for blood testing and clinical data collection. All patients were admitted to the Child Neuropsychiatric Unit of the Bellaria Hospital (IRCCS, Bologna) for a clinical diagnostic assessment and a comprehensive neurological work up. Autism diagnosis was made according to the currently accepted clinical evaluation panels^40^.

Parents were questioned regarding the age of onset of early autistic signs. In all 21 patients any medical, genetic and neurological comorbidity was excluded. In particular, chromosomal microarray analysis showed that children were negative for *de novo* Copy Number Variations. Patient total CARS scores ranged from mild to severe and developmental levels varied from normal IQ to severe cognitive impairment (Supplementary Table 1).

Control group children were healthy typically developing children, recruited in the local community, with no sign of cognitive, learning and psychiatric involvement, as clinically and anamnestically determined by experienced clinicians. All control group children were attending mainstream school and had not been subjected to stressful events. Dietary habits have been assessed by a food questionnaire. All patients and controls were on a typical Mediterranean diet. Both ASD and control groups were drug and supplementation free.

### HDFM equipment settings and spectral mapping of RBC

Dark-field images were recorded by using an enhanced dark-field illumination system (CytoViva, Auburn, AL) attached to an Olympus microscope (EDFM). The system consisted of a CytoViva 150 dark field condenser in place of the microscope original condenser attached *via* a fiber optic light guide to the lamp source. A 100x oil immersion colour corrected objective (Olympus UPlanAPO fluorite, N.A. 1.35–0.55) was integral to the system. A 150 W quartz halogen light source (Dolan Jenner DC-950, Massachusetts, USA) was used, which covers the full spectrum from 400 nm to 2500 nm. The human blood samples were examined following a protocol described in detail elsewhere^18^. Briefly, 5 μL of EDTA-treated whole blood were loaded in the centre of the slide and sandwiched with coverslip; this sample was left 120 minutes in order to avoid image blurring, and then optical acquisition started. From an optical image made of 700000 pixels, after background subtraction, the region of interest (ROI) was selected and the hyperspectral image of the sample was acquired with a resolution of 2 nm in the wavelength window of visible near-infrared (VNIR, 400 nm–1000 nm).

For reproducibility it was also established that only RBCs are present in the image field, no other blood cells, and 30 regularly shaped RBCs are selected for the ROI acquisition; two acquisitions were carried out for each sample.

Eight spectra were individuated with optimal coverage of the optical image confirming the previously individuated spectral library^18^. Supplementary Information reports the experimental procedures and Supplementary Fig. 1S (panel A) shows the spectra of the HDFM spectral library for healthy children RBC. The intensity arbitrary units were left as in the original HDFM spectra. The Savitzky-Golay filtering method was then applied to smooth the original signal data. A second order polynomial was fitted using 10 data points. Signal smoothing was done using OriginPro 8 software (OriginLab, Northampton, MA).

Using the SAM (Single Angle Mapper) function, setting the maximum angle (radians) on single value and keeping the maximum angle (radians) at 0.1, eight spectra were individuated which satisfied the requirements of optimal coverage of the optical image and confirmed the previously individuated spectral library^18^. Supplementary Information reports the experimental procedures. The arbitrary units defining spectral intensity were left as in the original HDFM spectra. The Savitzky-Golay filtering method was then applied to smooth the original signal data. A second order polynomial was fitted using 10 data points. Signal smoothing was done using OriginPro 8 software (OriginLab, Northampton, MA).

The spectral distribution of the eight HDFM end-members in the control samples is shown in Supplementary Fig. 1S (panel A); in Supplementary Table 2 the spectra 3, 4, 5, 6 are reported as mean values ± standard error of the mean (SEM) of two independent acquisitions for each sample. The other four spectra, namely 1, 2, 7, 8, were found to have large errors therefore they are not reported in the Table, but only their total spectral distributions are reported for ASD and healthy children in the footnote. The optical imaging with spectral matching of the library in representative RBCs of both control and patient groups are shown in Fig. 1, left and right panel, respectively. Figure 2 in the main text reports the eight histograms with the error bars of the control (dark grey) and ASD (light grey) groups. The HDFM spectrum 4 is reported in Fig. 3, and its distribution between control and ASD subjects was significantly different (Fig. 2 and Supplementary Table 2).

The spectra of phospholipids from egg lecithin and protoporphyrin IX, used as representative components of the RBC membrane and sub-membrane regions, were also acquired following previously reported procedures^18^, with further details in Supplementary Information. Their spectra are reported in Fig. 3 (together with the spectrum 4) as well as in Supplementary Fig. 1S (panel B).

Supplementary Table 3 reports the values of relative distribution percentages of the 8 HDFM spectra in healthy adult, as previously reported^18^, in order to evaluate the new data obtained in healthy children.

All measurements were reproducible after several hours from the sample preparation, the spectra and values of spectral distribution being similar in the range of ±1% up to 5 hours after the first acquisition.

### Membrane fatty acid analysis

An aliquot of the blood samples (250 μL) used for the HDFM measurements was then used for the separation of the blood cells, isolation of mature RBC membrane phospholipids and examination of fatty acid composition, applying previously published protocols^7^. We evaluated the fatty acids of the mature erythrocyte membrane phospholipids as relative percentages (% rel) referred to a cluster of 12 fatty acids (10 cis and 2 trans fatty acids)^2^. The mean % rel ± standard deviation (sd) of the RBC membrane fatty acid cluster for healthy and ASD children with the decreased value of DHA in the ASD children (*P* = 0.0424) are reported in the Supplementary Table 4.

### Statistical analysis

To compare groups, normality tests were applied to all numeric variables, following which appropriate parametric tests (ANOVA, Student’s *t* test for independent data) or the nonparametric equivalent (Wilcoxon-Mann-Whitney) were used. Non-parametric correlation (Spearman’s rho) was used to correlate clinical features and biochemical data in the ASD group (non-parametric ANOVA for cognitive/developmental level). Differences were considered significant at *P* value < 0.05. Spectrum 4 and DHA value were found statistically significant by ANOVA test with *P* values of 0.0021 and 0.0344, respectively (Supplementary Table 5). ROC (Receiver Operating Characteristic) curves were also used to estimate the performance of a given parameter as a binary classifier, *i*.*e*. of the ability of a test to assign a specific subject to either one of two groups – in this case healthy controls (negative group) or ASD patients (positive group). Plotting the true positive rate, or sensitivity, against the false positive rate, or specificity, at various threshold settings^19^, creates the curve. The sensitivity is the ability to correctly classify positive cases (patients), whereas the specificity predicts the ability to classify negative cases (controls). The ROC curves are here used in conjunction with the Youden Index (J), as a way of summarizing the performance of the diagnostic test. Its value ranges from 0 to 1, a value of 1 indicating the absence of false positives or false negatives. ROC curve analyses were based on non-parametric methods. The confidence intervals of ROC curves were set at 95% (Supplementary Figs 2, 6 and 7). When two parameters are combined, such as in the case of HDFM data and DHA value, a mathematical treatment to eliminate the measurement unit has been applied, as explained in Supplementary Information.

Principal components analysis (PCA) is a statistical method that uses an orthogonal transformation in order to sort out affinities among different variables; the higher the value of “factorial weights”, the higher the affinity of a variable to that specific factor. It was carried out for the different variables reported in this study finding four factors that gather >90% of the variability of the data. Factor 2 contains DHA values and spectrum 4 together with “cc”, which is the variable that discriminate cases and controls, and the CARS total score (Supplementary Fig. 8 and Table). Therefore, PCA indicates that these four parameters belong to the same factor, putting clinical, biophotonic and molecular data together with the variable that discriminates cases and controls.

Statistical analysis was performed using SAS v. 9.2 and STATA 12.

## Electronic supplementary material

Supplementary Information
