# Supplementary material for: High predictive values of RBC membrane-based diagnostics by biophotonics in an integrated approach for Autism Spectrum Disorders
Source: Sci Rep. 2017 Aug 29;7:9854. doi: 10.1038/s41598-017-10361-7 (PMC5574882; doi:10.1038/s41598-017-10361-7)
Supplement: Supplementary file 1 — Supplementary Information [file 41598_2017_10361_MOESM1_ESM.pdf]

Supplementary Information

High predictive values of RBC membrane-based diagnostics

by biophotonics in an integrated approach for Autism Spectrum Disorders

Giorgia Giacometti, Carla Ferreri, Anna Sansone, Chryssostomos Chatgilialoglu, Carla Marzetti, Ellas Spyratou, Alexandros G. Georgakilas, Marina Marini, Provvidenza M. Abruzzo, Alessandra Bolotta, Alessandro Ghezzo, Renato Minguzzi, Annio Posar & Paola Visconti*

Corresponding author: carla.ferreri@isof.cnr.it

Table 1S. Demographic and clinical features of the ASD children group

No	Gender	Age (Month)	Cognitive/ developmental impairment	Ados Score	Cars total score	Cars activity level item score	Cars stereotypies (body use) item score	CARS verbal language (expressive) item score	CARS non verbal language item score	CARS total number of items whose score was ≥3
1	m	68	Severe	20	41.5	2.5	3	3	3	11
2	m	94	Severe	21	46	3.5	4	4	4	11
3	f	68	Normal IQ	20	43.5	2.5	3	3.5	3	10
4	m	93	Severe	20	48.5	3	4	3.5	3	12
5	m	82	Moderate	19	42	3	3	3	3	9
6	m	115	Severe	19	39.5	3	3	3	3	7
7	m	74	Severe	19	41	2.5	3	3	3	9
8	m	99	Moderate	16	35	2.5	2	3	3	3
9	m	85	Moderate	22	41	2.5	2	3	3	7
10	f	74	Severe	17	38	2	2.5	3	3	7
11	f	124	Normal IQ	21	33.5	2	2	2.5	2.5	4
12	m	129	Normal IQ	19	39	2	2.5	3	3	7
13	m	78	normal IQ	17	39	3	2.5	3	3	6
14	m	96	moderate	22	44	2.5	3	3	3	11
15	m	84	normal IQ	18	39.5	2.5	3	3	3	8
16	m	114	severe	21	47	3	3.5	3.5	3	13
17	m	103	moderate	16	41.5	4	2	3	3	10
18	m	131	normal IQ	18	35	2	3	2.5	2.5	3
19	f	70	moderate	18	43	2.5	3	3	3	11.0
20	f	64	normal IQ	17.0	39.5	2.5	2.5	3.0	3.0	6.0
21	f	64	normal IQ	17.0	41.0	2.5	2.5	3.0	3.0	7.0

Hyperspectral-enhanced dark field microscopy (HDFM)

RBCs were examined in air and at room temperature by using an enhanced dark field illumination system (CytoViva, Auburn, AL) attached to an Olympus microscope (EDFM). The system consisted of a CytoViva 150 dark field condenser in place of the microscope original condenser, attached via a fiber optic light guide to a 150W quartz halogen light source (Dolan Jenner DC-950, Massachusetts, USA). The CytoViva dark field method picks up forward scatter from the sample at angles greater than 20 degrees from the forward light propagation, over a scattering cone of approximately 100 degrees. The angle of the forward incident light is approximately 70 degrees off of the normal to the sample slide, so as to miss the aperture of the objective lens. The scattering is isotropic in the plane of the sample slide. The angle is such that the direct illumination does not enter the objective lens, rendering a dark field. Therefore the sensitivity of this angular configuration is high, since the scatter is superimposed on a dark background.

With Cytoviva, complete Koehler illumination and a main feature of Critical illumination are achieved using a novel illumination system. Koehler illumination is pre-aligned in the device by fixing the light source precisely on the entrance slit of the condenser. This allows the user to adjust a focus point on the sample, which is a useful feature of Critical illumination, and is achieved when

the condenser is aligned with the objective to find the focal point on the sample. Thus, Koehler illumi- nation is initially fixed, and then CytoViva can be adjusted (up or down) to find the proper position and size of an illuminated spot for Critical illumination.

Scattering intensity strongly depends upon the light wavelength, thus corrections for image distor- tions are indispensable to achieve the highest possible resolution. The objective lenses used in the CytoViva microscope are color-corrected over visible wavelengths and in particular over the 450 – 650 nm range where hemoglobin absorption produces a spectral signature. The other components of the system, including microscope and spectrograph, are similarly corrected. Moreover, any chromatic aberration from the condenser is constant since the illumination path and angles of light

are pre-aligned. Other specifications can be found at the web address: http://cytoviva.com/wp-content/uploads/2016/08/Spec-Sheet-CytoViva-Optical.pdf

HDFM spectral library of healthy children RBCs

EDTA-treated whole blood was used as described in Methods. **Figure 1S, panel A** shows the 8 end-members of RBC HDFM imaging for healthy children. In **Figure 1S panel B**, the HDFM spectra of the two molecular components, phospholipids and protoporphyrin are plotted with spectrum 4 before the smoothing procedure as described in the Methods (see also Figure 3 in the main text).

A

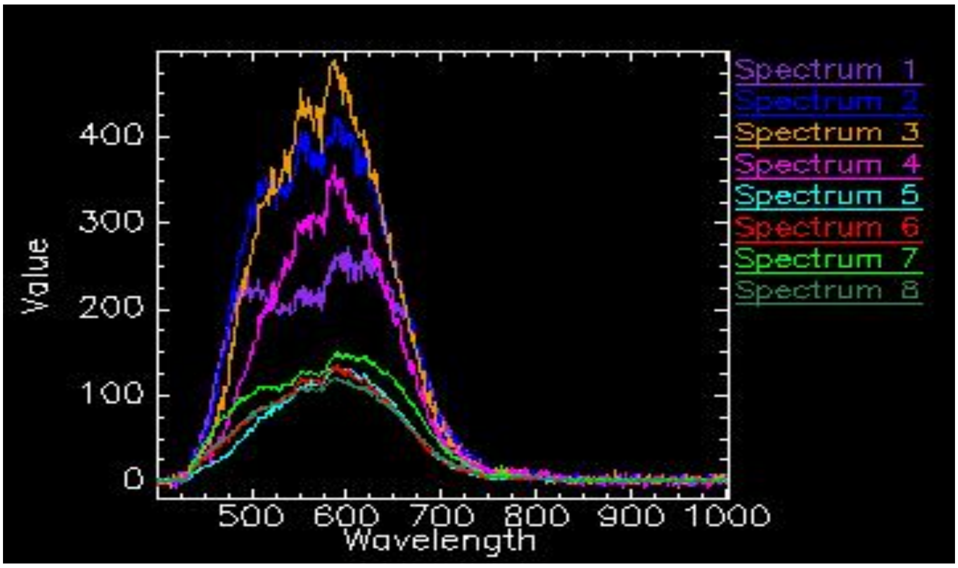

B

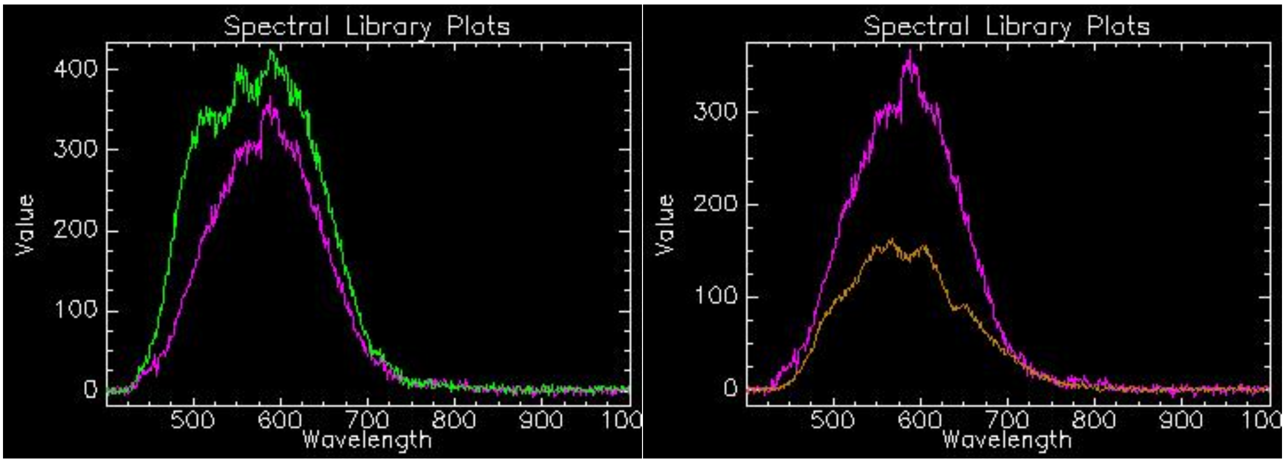

Figure 1S. Panel A: RBC HDFM spectral end-members of healthy children (first image).

Panel B: two representative compounds of the RBC membrane and sub-membrane regions prepared as described in Supplementary Information and ref. 1: phospholipids (left, green trace), protoporphyrin IX (right, brown trace) superimposed with the HDFM spectrum 4 (purple trace).

HDFM spectral library of RBC molecular components

HDFM spectral characteristics of representative compounds present in the molecular composition of the RBC membrane and sub-membrane region were reported previously [1]. Herein we report two compounds, whose spectral bands can be found in spectrum 4 of the RBC HDFM library. Biologically significant concentrations were used to prepare the samples as indicated in the literature for phospholipids [2] and protoporphyrin IX [3]. In particular, it is worth noting that when phospholipids are in aqueous system they spontaneously form liposomes vesicles, well known mimics of the membrane lipid organization.

Acquisition of the phospholipid HDFM spectrum

4.8 mM egg lecithin phospholipid suspension was prepared in tri-distilled H_2O according to known procedures [4]. Liposome vesicles were formed and the average vesicle diameter size was 200 nm as detected by dynamic light scattering (DLS) measurements (Zetasizer Nano Z, Malvern Instruments). From an optical image made of 700,000 pixel, after background subtraction, the region of interest (ROI) was selected and the hyperspectral image of the sample was acquired; the spectrum of phospholipid vesicles is shown in Figure 1S panel B (left, green trace) as well as reported in Figure 3 of the main text. Bands at 525, 563, 595, 620 nm are observed [1].

Acquisition of the protoporphyrin IX HDFM spectrum

6.5 mM protoporphyrin IX was dissolved in tri-distilled H_2O as previously described [1]. The biological concentration was chosen [3]. The procedure described above for phospholipids was followed, for the selection of the optical image and the region of interest (ROI) to obtain the

hyperspectral image of the sample; the spectrum of protoporphyrin is shown in Figure 1S panel B (right, brown trace) as well as reported in Figure 3 of the main text. Typical bands at 505, 555, 564, 605, 618 nm are observed as previously described [1].

Table 2S. Relative distribution of the HDFM spectral patterns of the RBC samples of healthy and ASD children (see Figure 2 in the main text).

Spectrum[§] #	Control children[*] (n=20)	ASD children[*] (n=21)	P values[¥]
3	7 ± 3	7 ± 2	0.6520
**4**	**13 ± 2**	**20 ± 3**	**0.0021**
5	53 ± 9	53 ± 6	0.9835
6	11 ± 3	8 ± 2	0.0883

[*]Two independent acquisitions for each sample were carried out. All the values are expressed as % rel ± SEM (standard errors of the mean); [¥] Student's *t* test (Aggregate correction for equal variances). [§]The numbering of the spectra is referred to the HDFM spectral library acquired for the human round-shaped RBC (ref. 1). The other four spectral members of the library (spectra 1-2-7-8) accounted for a total of 12% and 15% of the remaining spectral distribution in ASD and healthy children, respectively, and are not reported due to large errors detected in these children cohorts.

Table 3S. Values of HDFM spectral distribution (% rel ± SEM) of healthy adult control as previously reported[1]

Spectrum #	Adult controls (n=30)
1	1 ± 0
2	3 ± 0
3	7 ± 0
4	12 ± 2
5	54 ± 2
6	11 ± 1
7	7 ± 0
8	5 ± 0

Table 4S. Relative percentages (% rel ± sd) of the cluster of 12 fatty acids representing the main RBC membrane phospholipid hydrophobic components, evaluated as fatty acid methyl esters (FAME) by gas chromatographic analysis.

FAME	CTR (n=20)	ASD (n=21)	*P* values
Palmitic 16:0	24.8 ± 1.3	25.2 ± 0.9	0.7557
Stearic 18:0	18.2 ± 0.6	18.2± 0.7	0.8711
Palmitoleic 16:1	0.3 ± 0.04	0.3± 0.04	0.3549
Oleic 18:1	16.4 ± 0.3	17.1± 0.8	0.0960
Vaccenic 18:1	1.2 ± 0.1	1.2 ± 0.1	0.9350
Linoleic omega-6 18:2	12.8 ± 0.5	12.4 ± 0.6	0.2851
Eicosatrienoic omega-6 20:3	2.1 ± 0.2	2.1 ± 0.2	0.2973
Arachidonic omega-6 20:4	18.6 ± 1.3	18.6 ± 0.9	0.7660
EPA omega-3 20:5	0.6 ± 0.1	0.5 ± 0.1	0.4066
DHA omega-3 22:6	4.8 ± 0.6	4.1 ± 0.4	0.0424*
Trans 18:1	0.1 ± 0.0	0.1 ± 0.0	0.9443
Trans ARA	0.1 ± 0.0	0.1 ± 0.0	0.5415

Statistics

Here below are reported additional data and graphics on statistically significant values.

An odds ratio is defined as the ratio of the odds of an event occurring in one group to the odds of it occurring in another group. An odds ratio estimates the probability of disease given exposure to a specific factor by measuring the probability of exposure given the presence of disease. An odds ratio greater than 1 indicates that the event is more likely to occur in the group of exposure. An odds ratio less than 1 indicates that the event is less likely to occur in this group, and the factor of exposure is protective. An odds ratio equal to 1 means no differences between groups [5].

Table 5S. ANOVA TEST VALUES with DF= Degrees of Freedom and *t* value of the *t*-test statistic.

COMPARISON OF SPECTRUM 4 BETWEEN ASD AND HEALTHY CONTROL CHILDREN		
**DF**	**t**	**P value**
40	-3.2886	0.0021
COMPARISON OF DHA CONTENT BETWEEN ASD AND HEALTHY CONTROL CHILDREN		
**DF**	**t**	**P value**
39	-2.1937	0.0344

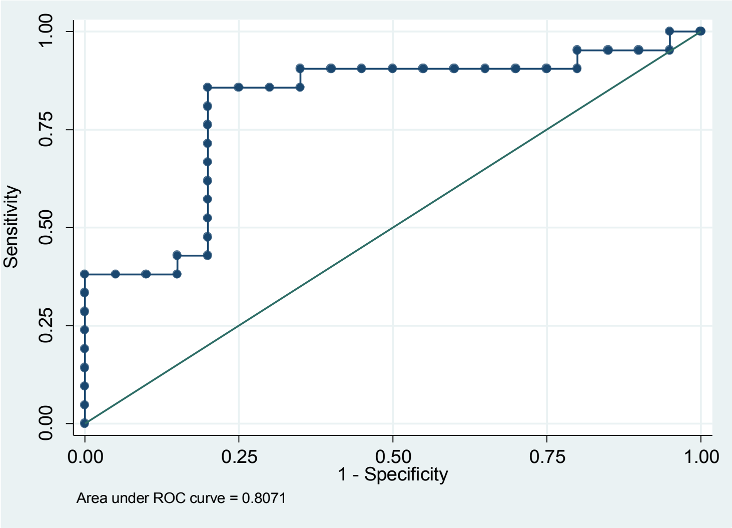

Figure 2S. ROC curves and J values for HDFM spectrum 4

**AUC**	**Cut off**	**Sensitivity**	**Specificity**	**J**
0.8071	16.225	85.71%	80.00%	0.6571

Statistical significance test of ROC curve yielded: z = |3.365|; P value= 0.0008. The cut off value has been used to classify spectrum 4 and include it as covariate in a logistic regression model whose outcome is the dummy variable that identifies cases and controls. The odds ratio was found to be significant and corresponding to 24 (P value <0.0001; IC 95%: [4.6488-123.9035]), which means that individuals with values of spectrum 4 higher than 16.225 have a probability of being autistic 24 times higher than those having values of spectrum 4 lower than 16.225.

CORRELATIONS

Spearman's non parametric correlations were calculated for spectrum 4 *vs.* a number of subject's demographic and clinical features. The correlations found to be statistically significant are shown.

Figure 3S. Correlation of HDFM spectrum 4 and CARS

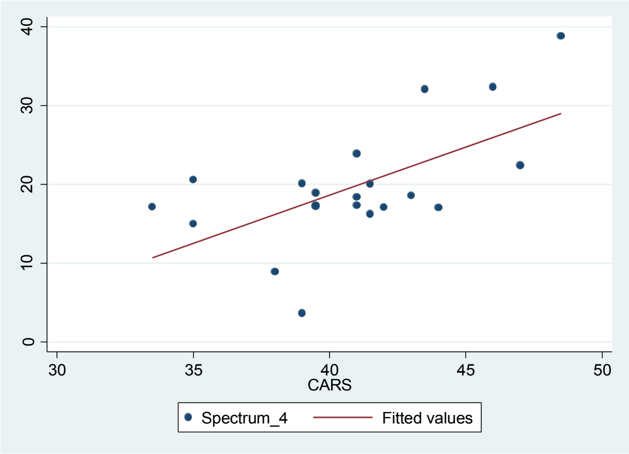

**Variable**	**Spearman's *rho***	**P value**
CARS	0.4881	0.0248

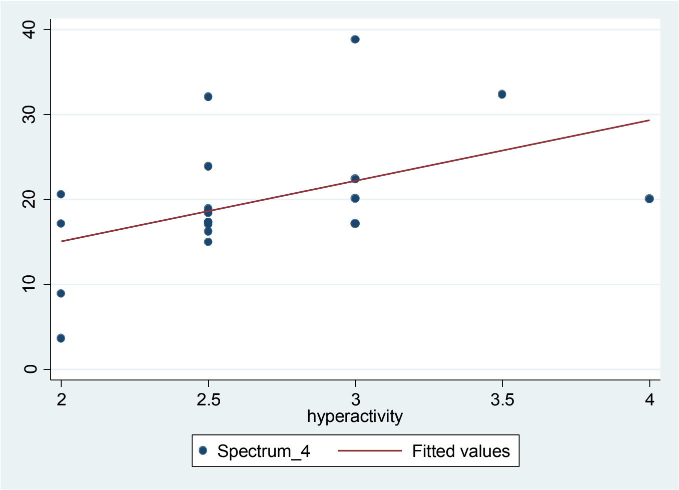

Figure 4S. Correlation of HDFM spectrum 4 and hyperactivity

**Variable**	**Spearman's *rho***	**P value**
Hyperactivity	0.4794	0.0279

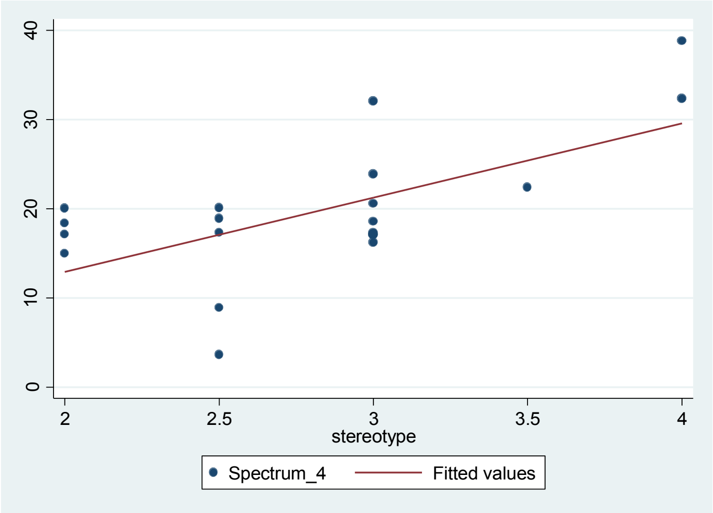

Figure 5S. Correlation of HDFM spectrum 4 with stereotype

**Variable**	**Spearman's *rho***	**P value**
Stereotype	0.4950	0.0225

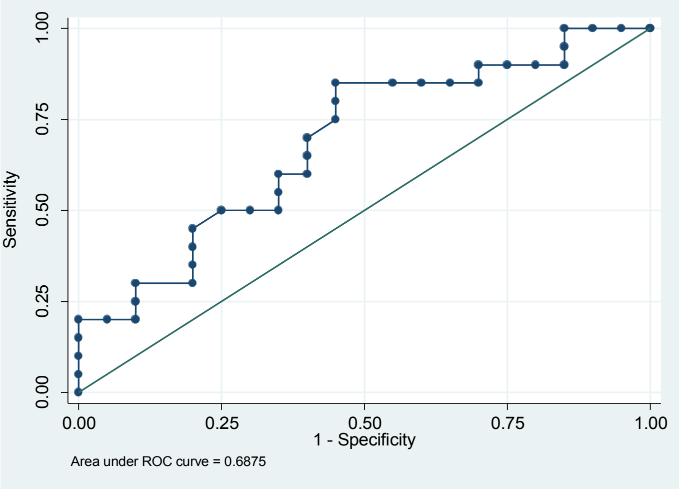

Figure 6S. ROC curve and J index for DHA

**AUC**	**Cut off**	**Sensitivity**	**Specificity**	**J**
0.6875	4.08094	85.00%	55.00%	0.4000

Statistical significance test of ROC curve yielded: z= |2.029|; P value= 0.0424. The odds ratio was found to be significant and corresponding to 6.23 (P value= 0.017; IC 95%: [1.3956-27.8412]), which means that individuals with values of spectrum 4 lower than 4.08 have a probability of being autistic 6.23 times higher than those having values of spectrum 4 higher than 4.08.

Combined ROC curve of HDFM spectrum 4 and DHA values

The ROC curve using both HDFM spectrum 4 and DHA values yielded the graphics shown in Fig. 6S. To evaluate the possibility to combine these two variables, each variable has been standardized using the following calculation: (xi - μ)/σ, where xi represent each observation from a variable, μ is mean value of that variable and σ the standard deviation. Thus we obtained z scores, that have no upper region and it is possible to add HDFM and DHA.

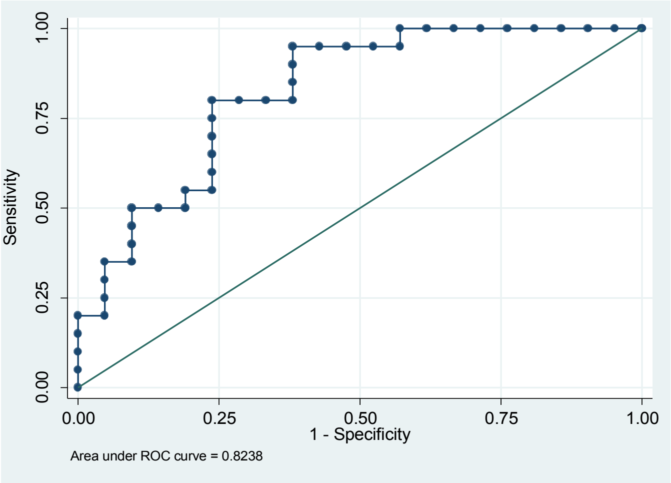

Figure 7S. Combined ROC curves and J index for the combination of HDFM spectrum 4 and DHA.

**AUC**	**Cut off**	**Sensitivity**	**Specificity**	**J**
0.8238	-0.4313	95.00%	61.90%	0.5690

Statistical significance test of the combined ROC curve yielded: z= |3.547|; P value= 0.0004.

The odds ratio was found to be significant and corresponding to 14.625 (P value= 0.002; IC 95%: [2.65624-80.52379]).

PRINCIPAL COMPONENT ANALYSIS

Principal component analysis is a statistical method that uses an orthogonal transformation in order to sort out affinities among different variables. The higher the value of "factorial weights", the higher the affinity of a variable to that specific factor. Four factors that gather the 90% of the variability of the data were individuated. Factor 1: 35%; Factor 2: 26%; Factor 3: 15% and Factor 4: 13%. Spectrum 4 was the only HDFM spectrum that belongs to the same factor (i.e., Factor number 2) that contains the variable "cc", which identifies cases and controls. It also contains DHA and CARS (Fig. 8S). The high value of the factorial weights associated to the variable "cc" (0.924) means that this factor is mainly determined by that variable. The "minus" sign of DHA means that the value "DHA" is higher with lower values of other variables belonging to the same factor. In the PCA analysis it is not considered the correlation between the elements of the analysis, therefore it is not expected that two factors that have affinity have also statistically significant correlation. The presence of age in the same factor is a confounding factor, due to the not-so-perfect match of ASD patients and controls.

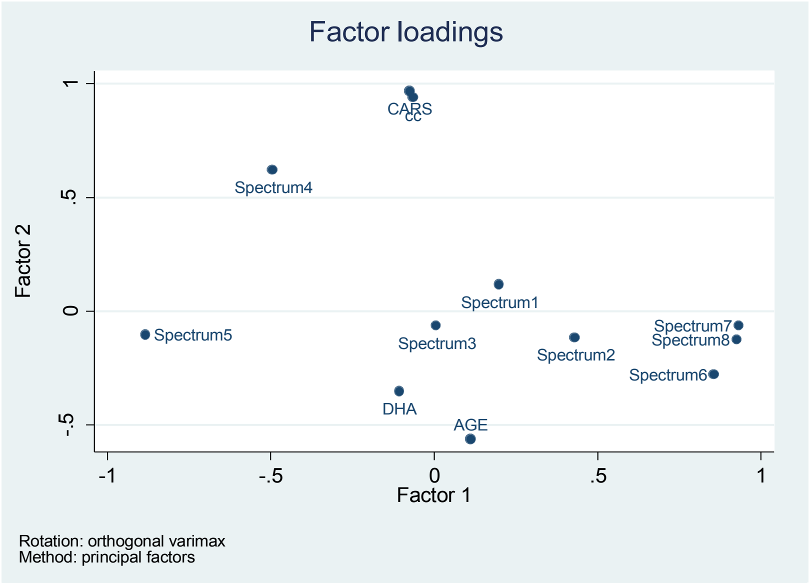

Figure 8S. Principal component analysis of the HDFM spectra and of relevant subject's parameters. In the Factor Loading Pattern only Factors 1 and 2 are shown, since they account for most of the variance.

In the Table the grey color highlights associations among parameters.

	Factor 1	Factor 2	Factor 3	Factor 4
**Spectrum 1**	0.196	0.122	0.083	0.908
**Spectrum 2**	0.444	-0.095	0.571	0.573
**Spectrum 3**	0.016	-0.046	0.962	0.072
**Spectrum 5**	-0.886	-0.110	-0.363	-0.152
**Spectrum 6**	0.871	-0.239	0.300	0.079
**Spectrum 7**	0.930	-0.067	-0.072	0.222
**Spectrum 8**	0.927	-0.141	-0.253	0.060
**Spectrum 4**	-0.502	0.606	-0.021	-0.361
**cc**	-0.075	0.924	-0.048	0.100
**Age**	0.141	-0.531	0.027	-0.072
**DHA**	-0.162	-0.455	0.264	-0.242
**CARS**	-0.084	0.956	-0.020	0.033

References

1- Conti, M., Scanferlato, R., Louka, M., Sansone, A., Marzetti, C. & Ferreri, C. Building up spectral libraries for mapping erythrocyte membrane by hyperspectral dark microscopy. *Biomed. Spectrosc. Imaging*, **5**, 175–184 (2016).

2- Nikolić, M., Stanić, D., Antonijević, N. & Niketić, V. Cholesterol bound hemoglobin in normal human erythrocytes: A new form of cholesterol in circulation? *Clin. Biochem.* **37,** 22–26 (2004).

3- Froom, P., Kristal-Boneh, E., Benbassat, J., Ashkanazi, R. & Ribak, J. Predictive value of determinations of zincprotoporphyrin for increased blood lead concentrations, *Clin. Chem.* **44,** 1283–1288 (1998).

4- Ferreri, C., Sassatelli, F., Samadi, A., Landi, L. & Chatgilialoglu, C. Regioselective cis-trans isomerization of arachidonic double bonds by thiyl radicals: the influence of phospholipid supramolecular organization. *J. Am. Chem. Soc.* **126**, 1063-1072 (2004).

5- Rothman, K.J. *Epidemiology: an introduction*, Oxford University Press, 2nd ed, 2013.
